# Supplementary material for: Reconciling Mining with the Conservation of Cave Biodiversity: A Quantitative Baseline to Help Establish Conservation Priorities
Source: PLoS One. 2016 Dec 20;11(12):e0168348. doi: 10.1371/journal.pone.0168348 (PMC5173368; doi:10.1371/journal.pone.0168348)
Supplement: S1 Dataset — (ZIP) [file pone.0168348.s002.zip › Taxa/Serra Norte/SN_2013/N5S/Lista N5S-07.pdf]

CAVIDADE N5S-0007

Classe	Ordem	Fam/Outros	Gên/Outros	Espécie	S-U	U-S	Zon.
Arachnida	Acari	Asternoseiidae		sp.84	X		E
Arachnida	Acari	Eupodidae		sp.1	X		P
Arachnida	Acari	Mesostigmata		jovem	X		E
Arachnida	Acari	Podocinidae	*Podocinum*	sp.1	X		P
Arachnida	Acari	Rhagidiidae		sp.20	X		E/P
Arachnida	Acari	Trombidiformes		sp.49	X		P
Arachnida	Acari			jovem		X	
Arachnida	Amblypygi	Phrynidae	*Heterophrynus*	*longicornis*	2	X	E
Arachnida	Araneae	Araneidae	*Alpaida*	aff. *negro*	X		E
Arachnida	Araneae	Caponiidae		sp.1	X	X	P
Arachnida	Araneae	Ctenidae		jovem	2		E
Arachnida	Araneae	Drymusidae		jovem	X		E
Arachnida	Araneae	Mysmenidae	*Microdipoena*	sp.1	X		E
Arachnida	Araneae	Ochyroceratidae	*Ochyrocera*	sp.3	X	X	E/P
Arachnida	Araneae	Ochyroceratidae	*Speocera*	sp.1	X		E/P
Arachnida	Araneae	Oonopidae	aff. *Xyccarph*	sp.1	X		P
Arachnida	Araneae	Pholcidae	*Leptopholcus*	sp.1	X		E
Arachnida	Araneae	Pholcidae	*Mesabolivar*	sp.1	X		E
Arachnida	Araneae	Prodidomidae	*Lygromma*	sp.3	X		P
Arachnida	Araneae	Salticidae		sp.10	X		E
Arachnida	Araneae	Segestriidae	*Ariadna*	sp.2	X		E
Arachnida	Araneae	Theraphosidae	aff. *Holothele*	sp.1	2		E
Arachnida	Araneae	Theridiidae	*Theridion*	sp.1	X		E
Arachnida	Araneae	Theridiosomatidae	*Plato*	sp.1	X		P
Arachnida	Opiliones	Cosmetidae	*Roquettea*	*singularis*	5		E
Arachnida	Opiliones	Escadabiidae		sp.2	X	X	P
Arachnida	Opiliones	Stygnidae	*Protimesius*	*gracilis*	8		E
Arachnida	Palpigradi	Eukoeneniidae	*Eukoenenia*	sp.1	X		E/P
Arachnida	Pseudoscorpiones	Chernetidae	*Spelaeochernes*	sp.1	X		E/P
Arachnida	Pseudoscorpiones	Chthoniidae	*Pseudochthonius*	sp.2	X		P
Arachnida	Pseudoscorpiones	Chthoniidae		jovem	X	X	E
Arachnida	Ricinulei	Ricinoididae	*Cryptocellus*	*tarsilae*	10	X	E/P
Chilopoda	Scutigeromorpha	Pselliodidae	*Sphendononema*	*guildingii*	3	X	E/P
Diplopoda	Polydesmida	Chelodesmidae		sp.		X	
Diplopoda	Polydesmida	Fuhrmannodesmidae		sp.1	X		E
Diplopoda	Polydesmida	Pyrgodesmidae		sp.1	X	X	P
Entognatha	Collembola	Entomobryidae		sp.1	X		E
Entognatha	Collembola	Entomobryidae		sp.4	X		P
Entognatha	Collembola	Paronellidae		sp.4	X		P
Entognatha	Collembola	Sminthuroidea		sp.2	X	X	P
Entognatha	Diplura	Campodeidae		sp.1	X		E
Entognatha	Diplura	Japygidae		sp.3	X		P
Insecta	Archaeognatha	Meinertellidae		sp.1	X		E
Insecta	Blattodea			jovem		X	
Insecta	Diptera	Keroplatidae	*Euceroplatus*	sp.	X		E
Insecta	Diptera	Phoridae		sp.		X	
Insecta	Diptera	Psychodidae	Phlebotominae	sp.	X		E/P
Insecta	Diptera	Psychodidae	*Pifanomyia*	*gruta*		X	
Insecta	Diptera	Psychodidae	*Sciopemyia*	*sordellii*		X	

Insecta	Heteroptera	Dipsocoroidea		sp.	X		P
Insecta	Homoptera	Cixiidae		jovem	X	X	E/P
Insecta	Hymenoptera	Formicidae	*Brachymyrmex*	sp.1	X		E/P
Insecta	Hymenoptera	Formicidae	*Brachymyrmex*	sp.2	X		E
Insecta	Hymenoptera	Formicidae	*Camponotus*	sp.2	X		E
Insecta	Hymenoptera	Formicidae	*Pachycondyla*	sp.		X	
Insecta	Hymenoptera	Formicidae	*Paratrechina*	sp.		X	
Insecta	Hymenoptera	Formicidae	*Paratrechina*	sp.1	X		E/P
Insecta	Hymenoptera	Formicidae	*Paratrechina*	sp.2	X		P
Insecta	Isoptera	Termitidae	*Nasutitermes*	sp.	X		E
Insecta	Lepidoptera	Tineoidea		jovem		X	
Insecta	Lepidoptera			jovem	X		E/P
Insecta	Mantodea	Mantidae		sp.1	4		E
Insecta	Orthoptera	Phalangopsidae	*Paraclodes*	sp.	9		E
Insecta	Orthoptera	Phalangopsidae	*Phalangopsis*	sp.	15	X	P
Insecta	Psocoptera	Epipsocidae	*Epipsocus*	sp.2	X		E
Insecta	Thysanura	Nicoletiidae		jovem	X		E
Gastropoda	Pulmonata	Systrophiidae	*Happia*	sp.	X	X	
Malacostraca	Isopoda	Armadillidae		sp.1	X	X	E
Malacostraca	Isopoda	Scleropactidae	*Circoniscus*	*buckupi*	X		P
Malacostraca	Isopoda	Scleropactidae		sp.1	X	X	P
Nematoda				sp.1		X	
Amphibia	Anura	Strabomantidae	*Pristimantis*	cf. *fenestratus*	7		E/P
Mammalia	Chiroptera	Furipteridae	*Furipterus*	*horrens*	2		P
